# Lecture on Nasal Obstruction

**Published:** 1901-05

**Authors:** 


LECTURE ON NASAL OBSTRUCTION. By A. Marmaduke Shield, F.R.C.S., Surgeon to St. George's Hospital, London. Published by P. Blakiston's Son & Co. Philadelphia. Price, $1.50.

This work of 104 pages is a product of three Clinical Lectures delivered by the author in connection with the Throat Department of St. George's Hospital in the year 1900. It treats of a subject which in the last few years has been the main topic at the meetings of the numerous rhinological societies. The subject is an important one and what the author says is presented in a clear conservative manner. The lectures were naturally intended for those who were not thoroughly familiar with the subject of nasal obstruction, and consequently much of the contents of the book appears rather elementary to the older worker in rhinology. All the various conditions which produce nasal obstruction are discussed and the author's own views given in detail. One excellent feature of the book is the practical and clear manner in which every subject is treated. The cuts illustrating the various conditions are very good, but like all illustrations, the artist's work is much easier than the actual demonstration. The small brochure is exceedingly handy and can well be recommended. R.

